# Influence of implant distribution on the biomechanical behaviors of mandibular implant-retained overdentures: a three-dimensional finite element analysis

**DOI:** 10.1186/s12903-024-04146-4

**Published:** 2024-03-30

**Authors:** Xiaoling Liao, Ruitao Cao, Juan Zhong, Chunxia Chen, Shaoxia Pan

**Affiliations:** 1 Department of Prosthodontics, Tianjin Stomatological Hospital, School of Medicine, Nankai University, No. 75, Dagu North Road, Heping District, Tianjin, 300041 China; 2 Tianjin Key Laboratory of Oral and Maxillofacial Function Reconstruction, Tianjin, 300041 China; 3 Department of Prosthodontics , Peking University School and Hospital of Stomatology & National Center for Stomatology & National Clinical Research Center for Oral Diseases & National Engineering Research Center of Oral Biomaterials and Digital Medical Devices& Beijing Key Laboratory of Digital Stomatology & NHC Key Laboratory of Digital Stomatology & NMPA Key Laboratory for Dental Materials, No. 22, Zhongguancun South Avenue, Haidian District, Beijing, 100081 PR China; 4 Hospital of Stomotology, Guanghua School of Stomatology, Guangdong Provincial Key Laboratory of Stomatology, Sun Yat-sen University, No. 56 Lingyuan West Road, Yuexiu District, Guangzhou, 510055 China

**Keywords:** Implant overdenture, Distribution of implants, Three dimensional finite element analysis

## Abstract

**Objective:**

To assess stress distribution in peri-implant bone and attachments of mandibular overdentures retained by small diameter implants, and to explore the impact of implant distribution on denture stability.

**Methods:**

Through three-dimensional Finite Element Analysis (3D FEA), four models were established: three models of a two mandibular implants retained overdenture (IOD) and one model of a conventional complete denture (CD). The three IOD models consisted of one with two implants in the bilateral canine area, another with implants in the bilateral lateral incisor area, and the third with one implant in the canine area, and another in the lateral incisor area. Three types of loads were applied on the overdenture for each model: a 100 N vertical load and a inclined load on the left first molar, and a100N vertical load on the lower incisors. The stress distribution in the peri-implant bone, attachments, and the biomechanical behaviors of the overdentures were analyzed.

**Results:**

Despite different distribution of implants, the maximum stress values in peri-implant bone remained within the physiological threshold for all models across three loading conditions. The dispersed implant distribution design (implant in the canine area) exhibited the highest maximum stress in peri-implant bone (822.8 µe) and the attachments (275 MPa) among the three IOD models. The CD model demonstrated highest peak pressure on mucosa under three loading conditions (0.8188 Mpa). The contact area between the denture and mucosa of the CD model was smaller than that in the IOD models under molar loading, yet it was larger in the CD model compared to the IOD model under anterior loading. However, the contact area between the denture and mucosa under anterior loading in all models was significantly smaller than those under molar loading. The IOD in all three models exhibited significantly less rotational movement than the complete denture. Different implant positions had minimal impact on the rotational movement of the IOD.

**Conclusion:**

IOD with implants in canine area exhibited the highest maximum stress in the peri-implant bone and attachments, and demonstrated increased rotational movement. The maximum principal stress was concentrated around the neck of the small diameter one-piece implant, rather than in the abutment. An overdenture retained by two implants showed better stability than a complete denture.

## Background

Edentulism has an important adverse impact on patients’ mastication, pronunciation, esthetics and quality of life. With longer life expectancy, the number of elderly edentulous patients with severe resorption of residual alveolar ridge is increasing gradually. Although complete denture (CD) prostheses are available to edentate populations, conventional CD for patients with severely atrophic residual alveolar ridge often show compromised retention and stability. Most problems occur with the mandibular denture. Edentulous patients suffer from denture lacking of stability and retention, inability to chew hard or tough foods and, in some, the dentures move, which may cause pain, food impaction and loosening in a social context.

With the development of implant technology, the prostheses of edentulism has been greatly improved. Implant-supported mandibular dentures can provide superior retention and stability compared to conventional CD. Previous research have demonstrated that patient satisfaction significantly increases for up to five years [1]. Notably, individuals with atrophic jaws, as classified by the Cawood and Howell criteria, experienced substantial improvements in quality of life [2]. The role of primary implant stability is crucial, especially in challenging cases like those with atrophic edentulous mandibles. High levels of implant stability are essential for the long-term success of rehabilitations and in minimizing marginal bone loss [3–6]. Despite the higher costs and surgical requirements of implant-retained fixed prostheses, implant-supported mandibular overdentures (IOD) have become an important way of prosthodontic treatment for edentulous patients [7]. The overwhelming evidence in favor of implant overdentures leads to the consensus that mandibular two-implant overdentures should be considered “as the first choice standard of care for edentulous patients” [1, 8–11]. Nevertheless, there is still debate among clinicians regarding the optimal placement of the two implants. Some studies suggested positioning implants in the lateral incisor area, rather than in the canine area, could minimize hinge movement [12]. Others, however, dispute this connection [13]. The aim of this three-dimensional Finite Element Analysis (3D FEA) is to evaluate stress or stain distribution in the peri-implant bone and attachments, and to explore denture stability of mandibular overdentures retained by implants in different positions. We hypothesize that different implant distribution may influence the stress distribution in the peri-implant bone, attachments, and the biomechanical behaviors of the overdenture.

## Materials and methods

**Fig. 1:**
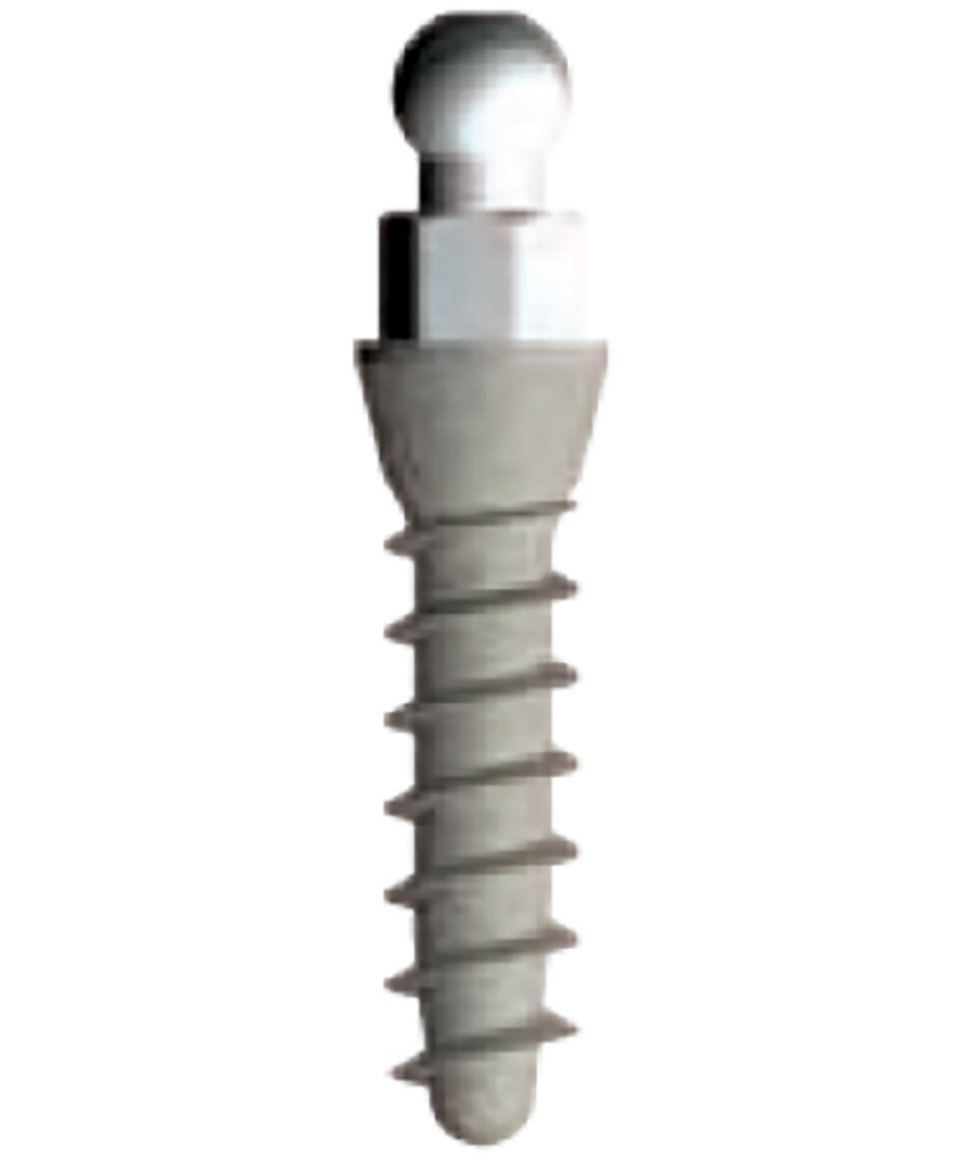
Tixos Nano OVD SDI and ball-attachment model

This study was performed by using 3D FE model analysis of a human mandible. An edentulous mandible and the complete denture of a typical female subject were chosen for the finite element (FE) model establishment. A computed tomography (CT) scan was conducted on this volunteer, with approval from the ethnical committee of Peking University School of Stomatology (IRB00001052-07051). The CT scan data was imported into Mimics8.0 (Materialise, Leuven, Belgium). The modeled section of the mandible consisted of a cancellous core surrounded by a 2.0 mm thick cortical layer and a 2.0 mm thick mucosa layer. The Tixos Nano OVD small diameter implant (Leader Italia Corporation, 2.7 mm*10 mm) with Tixos ball attachment (Fig. 1) was selected as the overdenture retainer for this biomechanical analysis. The geometry of the individual implants and their attachments was modeled according to engineering drawings. The models were created using Solidworks 2008 (SolidWorks Corporation, Vélizy-Villacoublay, France) and ABAQUS/CAE 6.8 (Simulia Corporation, Vélizy-Villacoublay, France).

In the absence of information concerning the precise organic material properties of bone, cortical and cancellous bone was treated as isotropic, homogenous, and linearly elastic, similar to the other materials utilized in this analysis. The implants were rigidly attached along their entire interface and bonded in the bone to simulate 100% osseointegration. Boundary fixations included constraining all three degrees of freedom at each of the nodes located at the most external mesial or distal aspects of the model. The mechanical parameters of the materials in this study was shown in Table 1.

**Table 1:** The mechanical parameters of the materials in this study

Type of material	modulus of elasticity (MPa)	Poisson’s ratio [14]
Ti-6Al-4V	114,000	0.35
Cortical bone	13,700	0.3
cancellous bone	1370	0.3
mucosa	1	0.37

**Fig. 2:**
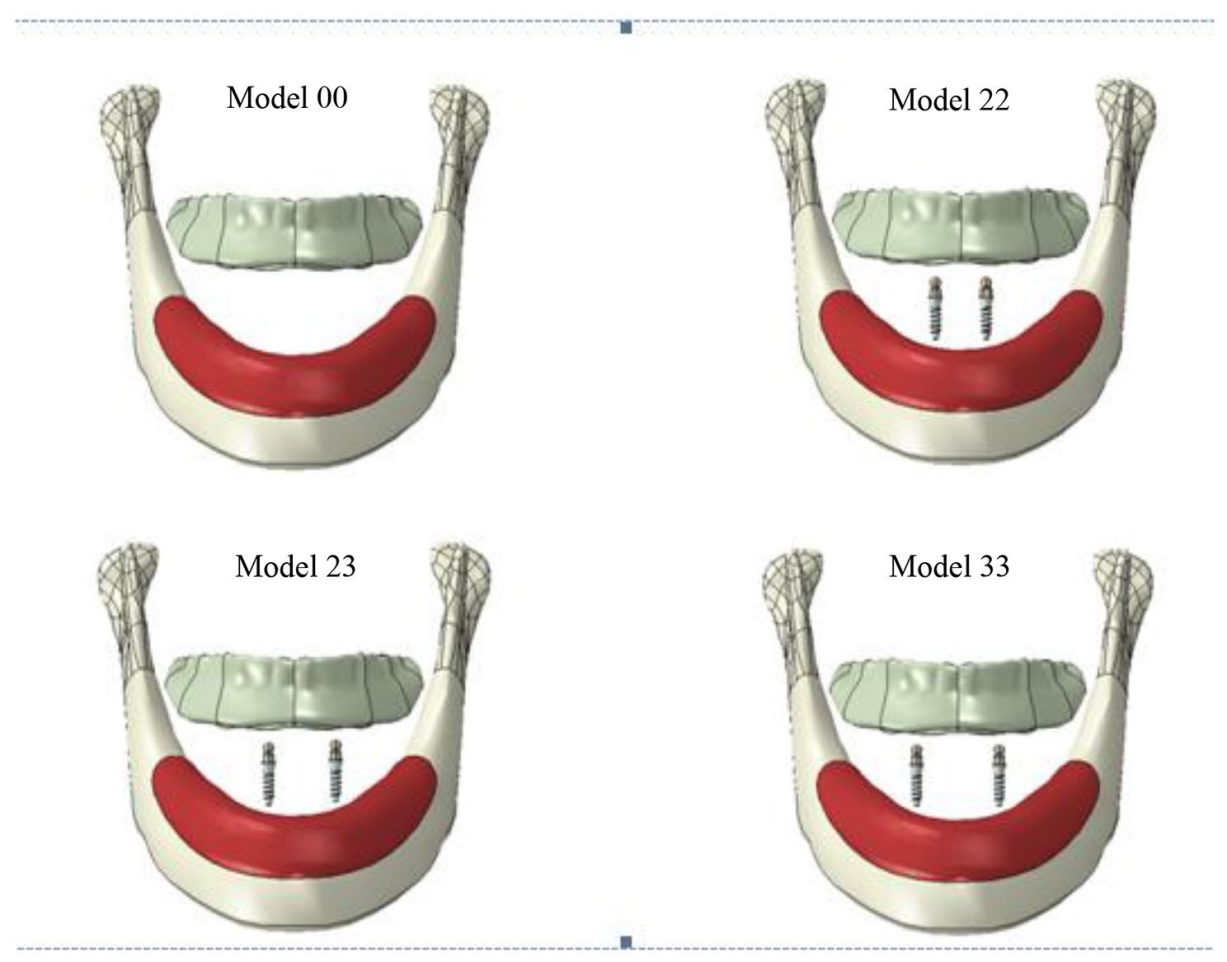
The diagram of the three-dimensional finite element models

Four models were established: three models of two mandibular implants retained overdenture ( IOD model) and one model of conventional complete denture (CD model) (Fig. 2). The four models were as follows:

Model 00: Conventional complete denture.

Model 22: Two small diameter implants placed in the area of the bilateral lateral incisors with an inter-implant distance of 12 mm.

Model 23: One small diameter implant placed in the canine area and another in the lateral incisor area, with an inter-implant distance of 16 mm.

Model 33: Two small diameter implants placed in the area of the bilateral canines, with an inter-implant distance of 20 mm.

The IOD model consisted of the superstructure and the complete denture. All implants were vertically positioned and well distributed in the interforaminal region, maintaining a minimum distance of 6 mm mesial to the mental foramen. The interface between the overdenture and the mucosa was not fixed during function. Instead, the overdenture was able to rotate and slide on the mucosa in different directions. The sliding friction between the denture and mucosa was simulated with a friction coefficient of 0.334 [15].

**Table 2:** The number of elements and nodes in five models

	Number of elemets	Number of nodes
Model 00	43,847	9173
Model 22	126,723	48,032
Model 23	127,968	48,255
Model 33	126,896	48,061

**Fig. 3:**
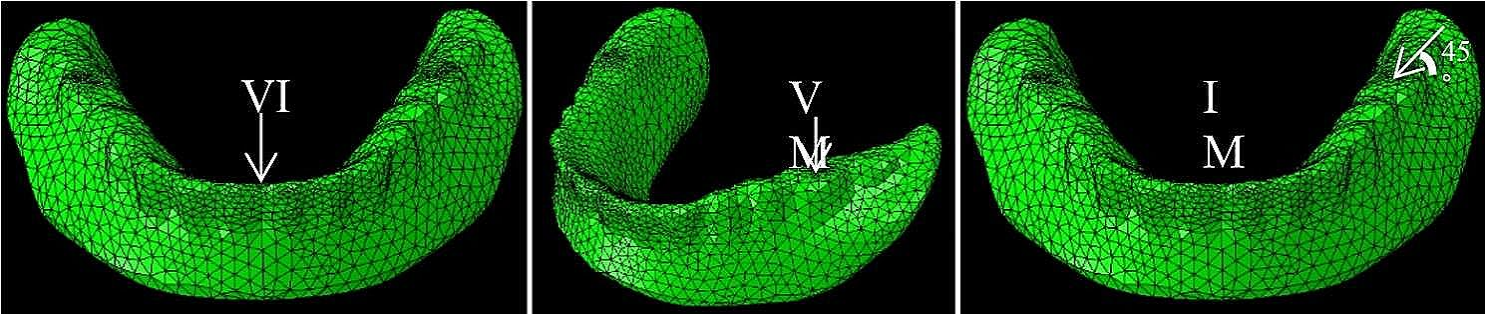
The three types of load applied on the denture

The models were meshed with 3D four-node tetrahedron elements. The number of the elements and nodes was detailed in Table 2. To accurately replicate the complex stress distribution observed in peri-implant bone, a refined mesh was generated in the interforaminal region. The loading protocol adopted in the study was delayed. Three types of load were applied on the denture for each model to simulate functional loading: a 100 N vertical load (VM), an inclined load (IM) on the left first molar, and a 100 N vertical load on the lower incisors (VI), as indicated in Fig. 3. IM refers to a 45° angled force applied buccolingually applied at the centre of the left first molar. The stress distribution in the peri-implant bone, attachments, and biomechanical behaviors of the overdenture were recorded. The overall process was summarized in Table 3.

**Table 3:** The flow chart of this study

Study steps	Detailed items
Model establishment	1. Model 00
	2. Model 22
	3. Model 23
	4. Model 33
Type of load	1. VI
	2. VM
	3. IM
Results analysis	1. Stress distribution in peri-implant cortical bone
	2. Stress in abutments
	3. Pressure on the mucosa and the contact area between the denture and the mucosa
	4. Displacement of denture free end

## Results

### Stress distribution in peri-implant cortical bone

Under three types of load, the highest value for the principal stress was found in Model 33 under vertical incisal loading. The values were shown in Table 4.

**Table 4:** Maximum principal stress in peri-implant cortical bone under three loading conditions (µe)

Loading condition	Model 22	Model 23	Model 33
VM	159.9	165.9	433.3
IM	329.8	343.1	822.8
VI	135.8	152.1	298.5

### Stress in abutments

Under three types of load, the maximum principal stress in the abutments of Model 33 was higher than or comparable to those in Model 22 and 23. Accross the three IOD models, the maximum principal stress in the abutments under IM was higher than or comparable to those under VM and VI. as detailed in Table 5.

**Table 5:** Maximum principal stresses in abutments under three loading conditions (MPa)

Loading condition	Model 22	Model 23	Model 33
VM	82.6	93.9	91.6
IM	166	242	275
VI	158	175	192

### Pressure on the mucosa and the contact area between the denture and the mucosa

**Fig. 4:**
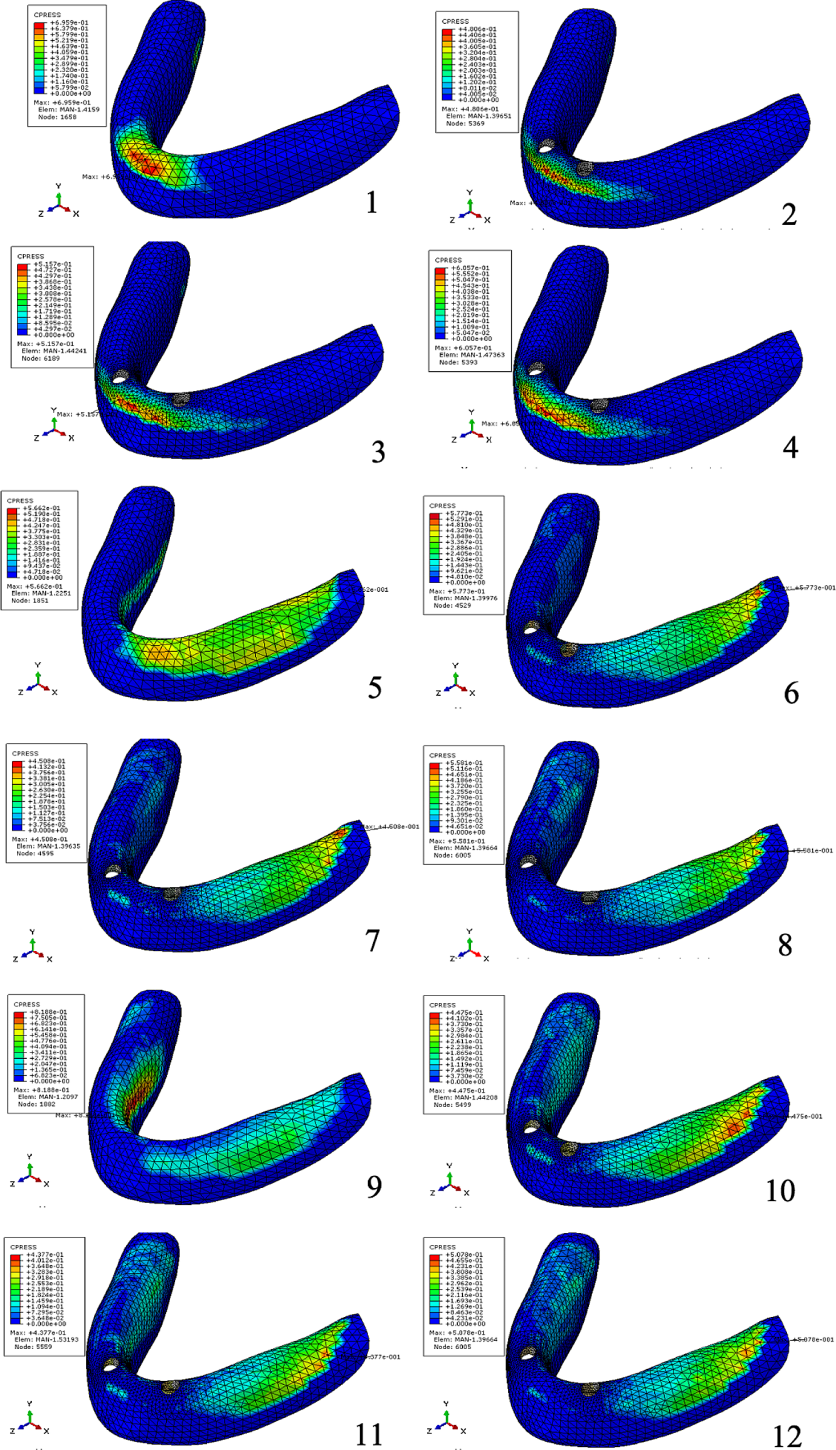
Distribution of maximum pressure on mucosa (1) in Model 00 under VI load, (2) in Model 22 under VI load, (3) in Model 23 under VI load, (4) in Model 33 under VI load, (5) in Model 00 under VM load, (6) in Model 22 under VM load, (7) in Model 23 under VM load, (8) in Model 33 under VM load, (9) in Model 00 under IM load, (10) in Model 22 under IM load, (11) in Model 23 under IM load, (12) in Model 33 under IM load

**Fig. 5:**
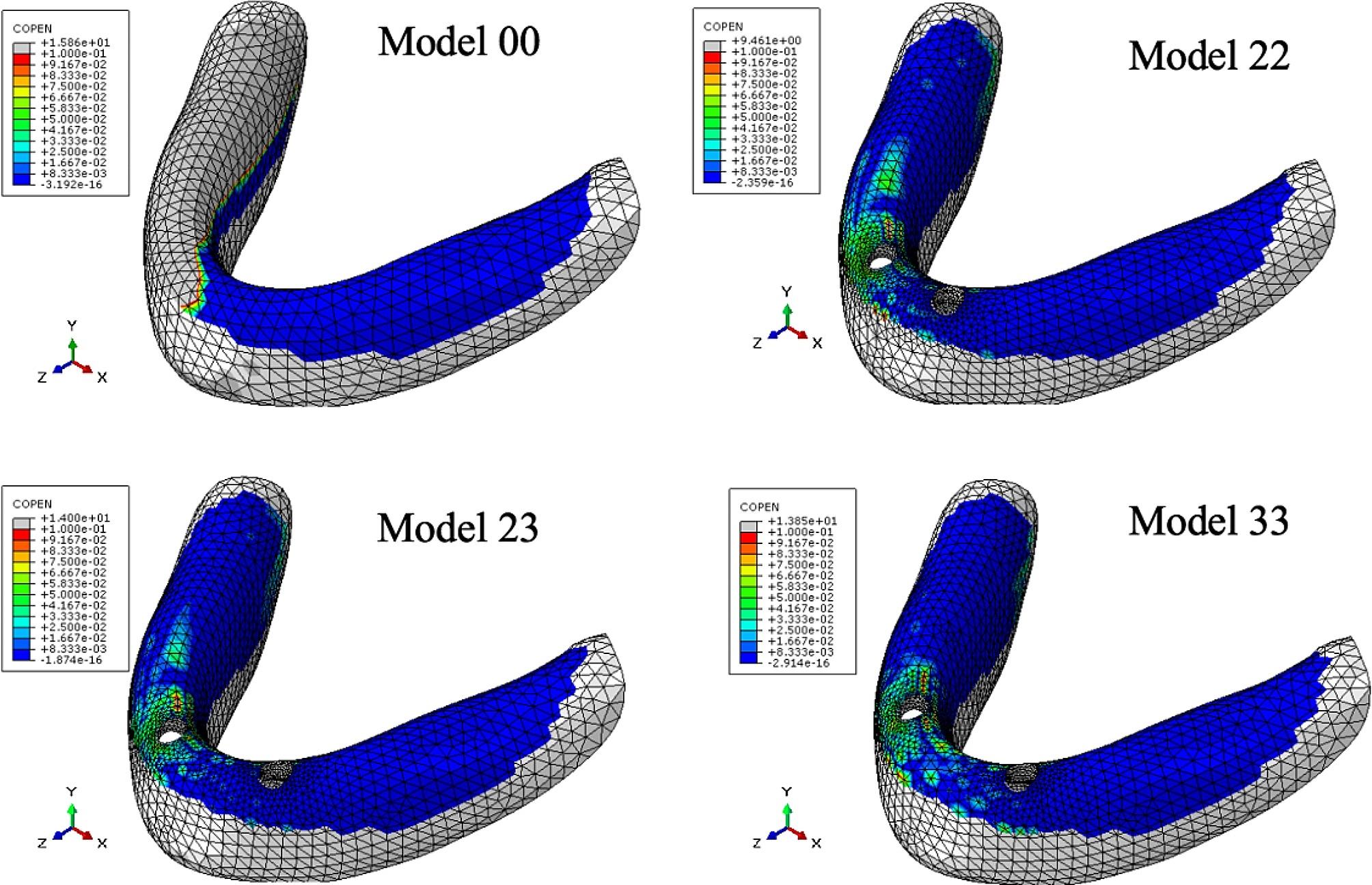
Distribution of contact area between the denture and mucosa under VM load. The cold tone represents the area where contact with the denture was close and tight, whereas the warm tone indicates the area where the denture tilted and separated from the mucosa.

**Fig. 6:**
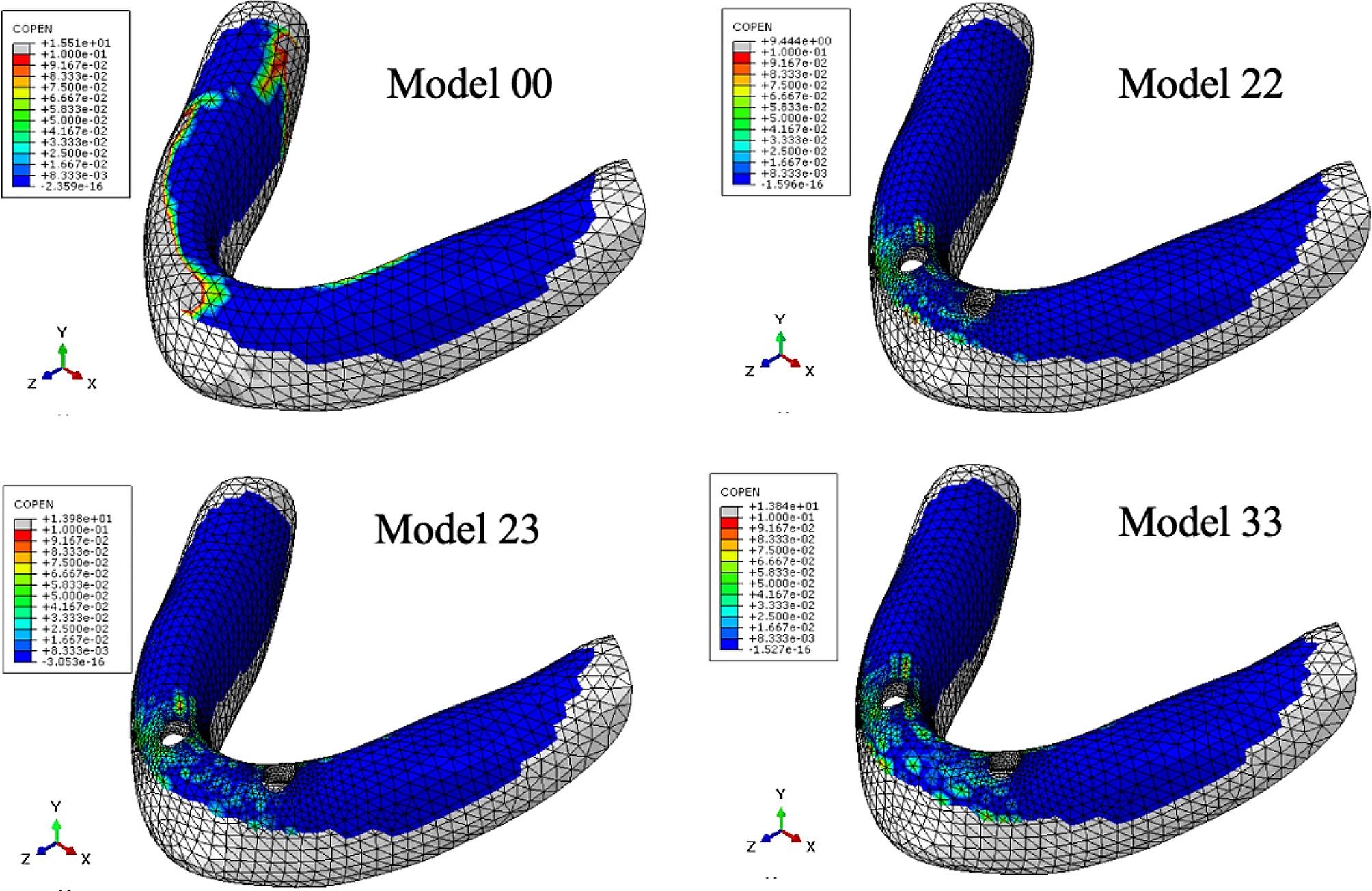
Distribution of contact area between the denture and mucosa under IM load

**Fig. 7:**
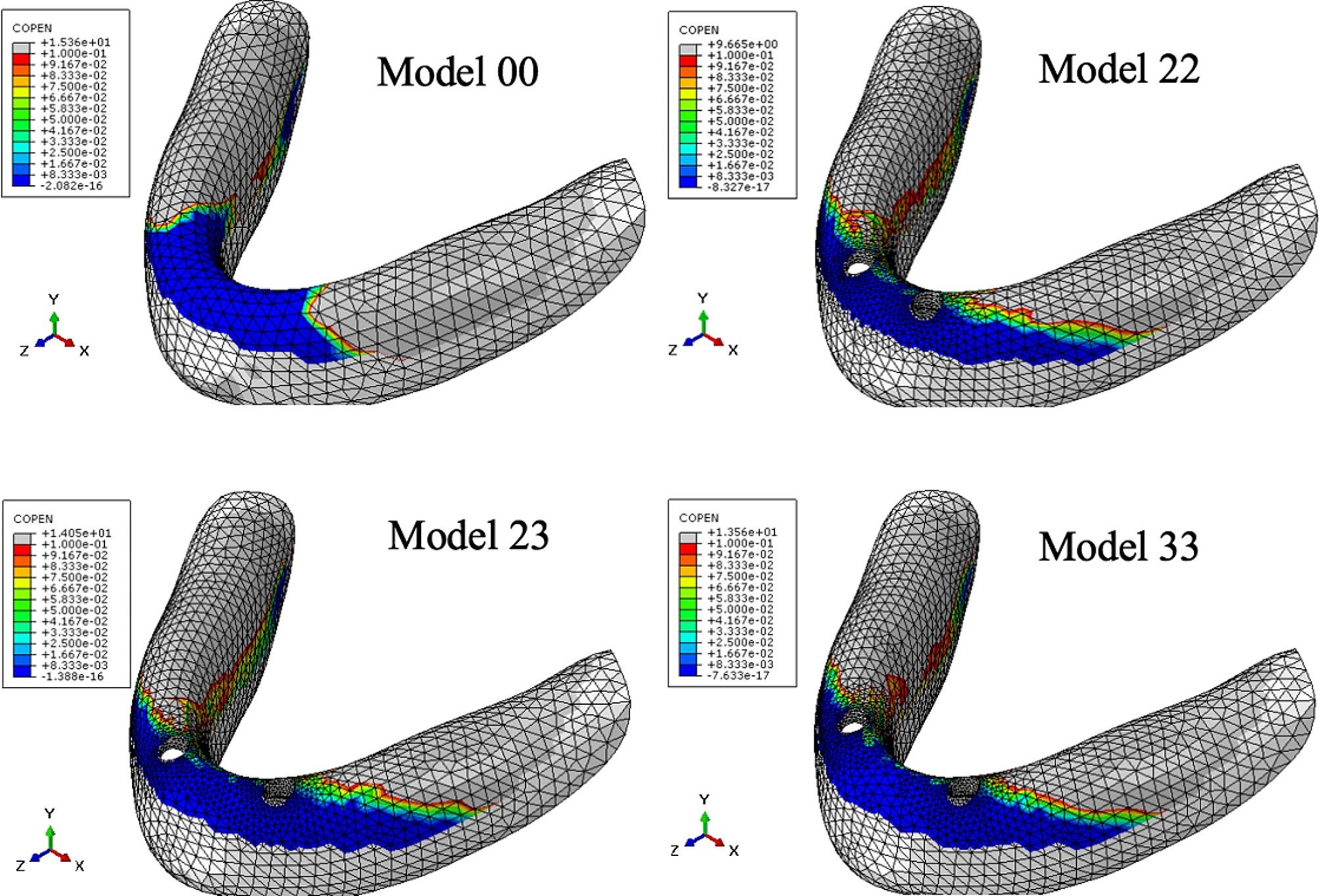
Distribution of contact area between the denture and mucosa under VI load

Table 6; Fig. 4 show the maximum pressure on the mucosa, while Table 7; Figs. 5, 6 and 7 display the contact area between the denture and mucosa. Under all three loading conditions, the CD model exhibited higher maximum pressure on the mucosa compared to the IOD models. The highest pressure was recorded in Model 00 under IM loading, specifically on the lingual side of the opposite posterior region. Among IOD models, the maximum pressure in Model 33 was higher than those in the Model 22 and 23 under VI loading, with the pressure concentrated between the labial side of the anterior alveolar ridge and the denture. Under VM and IM loading, the contact area between the denture and mucosa was more extensive than under VI loading. Additionally, under VM and IM loading, the contact area between the denture and mucosa in model 00 was smaller compared to the IOD models. Conversely, under VI loading, Model 00 exhibited a larger contact area. The contact area in IOD models remained consistent under VM and IM loading. Furthermore, the contact area in Model 33 was larger than in Model 22 and 23 under VI loading.

**Table 6:** Maximum pressure on mucosa under three loading conditions (MPa)

Loading condition	Model 00	Model 22	Model 23	Model 33
VM	0.5662	0.5773	0.4508	0.5581
IM	0.8188	0.4475	0.4377	0.5078
VI	0.6959	0.481	0.5157	0.6057

**Table 7:** Contact area between the denture and mucosa under three loading conditions (mm^2^)

Loading condition	Model 00	Model 22	Model 23	Model 33
VM	1100.11	1568.31	1587.07	1623.85
IM	1236.13	1552.74	1539.21	1521.23
VI	488.548	282.94	307.661	342.812

### Displacement of denture free end

Table 8 illustrates the displacement of the denture’s free end, with the majority occurring in the occlusal direction under VI load. Across all three loading conditions, displacement of denture free end in the CD model was greater than that in the IOD models, although the difference between the IOD models under the same loading conditions was minimal.

**Table 8:** Displacement of denture free end (mm)

Loading condition	Model 00	Model 22	Model 23	Model 33
VM	2.86	0.58	0.49	0.56
IM	1.27	0.73	0.69	0.74
VI	3.76	1.32	1.39	1.37

## Discussion

### Influence of implant distribution on the biomechanical behaviors of mandibular implant-retained overdentures

Under all three loading conditions, the maximum principal stress values were below 2500µe in all models [16], indicating that they were within the physiological tolerance threshold of bone. This suggests that all three distributions of two implants in our study are viable.

The highest value for the maximum principal stress in peri-implant cortical bone and stress in abutments were observed in Model 33 under three types of load. This may be attributed to the position of canine, which is located at the corner of the arch, leading to a stress concentration in cortical bone in this area. Furthermore, the greater the dispersion between the two implants, the closer they were to the posterior masticatory center, resulting in increased functional loading and consequently higher stress in peri-implant cortical bone. Both tensile stress and compressive stress were present in the cortical bone around the implants. It was noted that tensile stress values increased at the cervical region of the buccal side as the distance between implants grew, and decreased as the distance diminished. With shorter distance between implants, the compressive stress values increased and were located at the lingual surface of the cervical region [17]. The tensile and compressive stress in the cortical bone around the implant had counteracting effect when the inter-implant distance was 10 mm [18]. An inter-implant distance of 10 mm was identified as optimal for two fixture implantation, as it resulted in the minimum maximum stress in peri-implant cortical bone. In this study, the inter-implant distance for Model 33 was 20 mm, while it was 12 mm for Model 22. A more mesial placement of the implants resulted in a shorter distance between them, leading to reduced stress in the cortical bone surrounding the implant [19]. Lower tensile values were observed in cortical bone of a removable prosthesis supported by implants in the lateral incisor region [20].

The implant selected for this analysis was one-piece implant, which was not stress-broken. Consequently, the maximum principal stress on the abutments was concentrated around the cortical bone rather than within the abutments themselves. In three models, the maximum principal stress under IM in the abutments was higher than or comparable to those under VM and VI. The findings indicated that the horizontal load had a more significant inpact on the internal stress distribution of the abutments.

The attachment system played a important role in the success of implant overdenture. The risk of failure increased when the load was directly applied on the implant through a rigid connection. Therefore, it is essential for the abutments to be resilient, absorbing the force. In the case of two implant retained IOD, the overdenture rotated around the fulcrum line of the two ball abutment when loaded [21]. Among various IOD models, the maximum pressure was observed between the labial side of the anterior alveolar ridge and the denture, indicating that the overdenture rotated in anterior-posterior dimension. Sepcifically, in Model 22, the maximum pressure on the mucosa and the contact area between the denture and mucosa were minimal, indicating that under VI loading, positioning the implants more mesially results in reduced rotation of the overdenture, leading to decreased pressure on the mucosa and a smaller contact area between the denture and mucosa.Regarding maximum pressure on the mucosa, positioning the implants in the lateral incisor region had a beneficial effect under inclined loads.

Previous research showed the inter-implant distance of 27 mm provided better resistance to posterior dislodgment than placing two MDIs close together at 19 mm [22]. Two implants retained overdenture with implants placed in the area of the bilateral lateral incisors (inter-implant distance was 12 mm) may offer enhanced stability in our research. However, this study found minimal differences in the displacement of the denture’s free end among IOD models under the same loading condition in this study. This result was consistent with Kimoto’s clinical observation [21].

This study indicated that different implant distribution could influence the biomechanical behaviors of mandibular implant-retained overdentures. Implants placed more mesially resulted in lower maximum principal stress in the peri-implant cortical bone and reduced stress in abutments. This result was consistent with previous clinical observation which showed research a small inter-implant distance, more frontal, sagittal inter-implant divergence increased maintenance [1].

### Comparison between CD model and IOD models

There were significant differences in pressure on the mucosa, the contact area between the denture and the mucosa, and displacement of the denture’s free end between the CD model and IOD models. The greater the pressure on the mucosa, the higher the pressure on the mucosa of denture-bearing area, and the more mucosal supported the denture had. Under all three loading conditions, the maximum pressure on the mucosa in the CD model was higher than those in the IOD models. The mandibular complete denture was completely mucosal supporting, while implant-retained overdenture was supported by both the implant and mucosa.

The contact area between denture and mucosa indicated the degree of detachment between them. The less contact between denture and mucosa were, the greater the detachment. Under VM and IM, the contact area between the denture and mucosa in IOD models were similar, and was higher than those in the CD models. The implants stabilized the denture, preventing tilt, swing, and rotation during function, thus reducing denture detachment from the mucosa to some extent. Under VM and IM, the contact area between the denture and mucosa was larger than that under VI across all models, indicating more rotation of the denture under incisal loadiing than molar loading. Under VI, the contact between the denture and mucosa mainly occurred on the labial side of the anterior alveolar ridge, suggesting the denture rotated forward and backward [23]. Under VI the largest contact area was observed in the CD model, due to the subsidence of the anterior base of complete denture under incisor load. The denture pressed against labiolingual side of the anterior alveolar ridge of the mandible, resulting in a higher contact area in complete denture than in implant-retained overdentures. However, mandibular overdenture rotated along the fulcrum line, pressing against the labial side of the anterior alveolar ridge of the mandible. The more distally two implants were placed, the more rotation occurred.

Under all three loading conditions, the displacement of denture free end in the CD model was greater than those in the IOD models. This result indicated that implant retention could significantly improve the denture stability, and ball attachments offered some degree of resilience.

In summary, the implant-retained overdenture exhibited greater stability than the complete denture. From a biomechanical prespective, the implants effectively counteracted lateral loads, thereby minimizing denture rotation.

### Influence of unsymmetrical distribution of implants on the biomechanical behaviors of mandibular implant-retained overdentures

Under load on the incisor, the maximum principal stress, the maximum pressure on mucosa, and the displacement of the denture’s free end in Model 23 (unsymmetrical distribution of implant ) were all observed on the right side (more mesial side). The principal stress around the more mesially placed implant increased, the pressure on the mucosa concentrated on this side, and the displacement of the denture’s free end was more significant. This result indicated that the denture rotated along the fulcrum line. In clinical practice, when the implant positions were not symmetrical due to the limitation of the patient’s bone mass, the more mesially placed implant would receive more stress. The results suggested that clinician should pay closer attention to the implant on this side during follow-up visits, to prevent the adverse effect of unsymmetrical implant distribution on retention. Clinicians could select implants with a larger diameter [24–26] or longer length [27] during surgery, or connect the two implants rigidly with bar attachments [28] or use locator attachments as short as possible for more favorable stress distribution [29], or choose attachment types that allow rotation and can tolerate various angles [30].

### Other influence factors of denture stabilization

Rotational movement had a negative effect on perceived chewing ability. The stabilization of complete denture and implant-retained overdentures was associated with the height and shape of the residual alveolar ridge, the artificial teeth arrangement, the fit between tissue surface and denture base, the relationship of upper and lower arch, occlusal balance and neuromuscular coordination and so on. The anterior tooth arrangement and arch form were main influence factors. The arch form in this study was similar to oval or tip round, with the tooth arrangement positioned atop the crest of the alveolar ridge, thus enhancing denture stability [21]. Altering the tooth arrangement or selecting subjects with a square-round arch form could affect the biomechanical behaviors of the denture, and further studies are needed.

There were some limitations in this study. A series of assumptions and simplified methods were applied in this three-dimensional Finite Element Analysis, rendering the FEA models different from an actual patient’s jaw. Due to the lack of precise data on the organic material properties of bone, both cortical and cancellous bone was assumed to be isotropic, hemogenous and linearly elastic, similar to the other materials in this analysis. The modeled section of the mandible was composed as a cancellous core surrounded by 2.0 mm thick cortical layer and 2.0 mm thick mucosa layer. The implants were modeled to simulate 100% osseointegration, which may be inconsistent with actual conditions.

## Conclusion

IOD with implants in canine area exhibited the highest maximum stress in the peri-implant bone and attachments, and demonstrated increased rotational movement. The maximum principal stress was concentrated around the neck of the small diameter one-piece implant, rather than in the abutment. An overdenture retained by two implants showed better stability than a complete denture.

## Data Availability

The data used to support the findings of this study will be available from the corresponding author upon request.

## Abbreviations

3D FEA: Three Dimensional Finite Element Analysis
IOD: Implants retained overdenture
CD: Complete denture
CT: Computed tomography
VM: Certical loads on the left first molar
IM: Inclined loads on the left first molar
VI: Vertical loads on the lower incisors
